# Low-Stiffness Hydrogels Promote Peripheral Nerve Regeneration Through the Rapid Release of Exosomes

**DOI:** 10.3389/fbioe.2022.922570

**Published:** 2022-06-23

**Authors:** Zhixiao Liu, Hua Tong, Jian Li, Ling Wang, Xiaoyi Fan, Honghao Song, Mei Yang, Haowei Wang, Xin Jiang, Xuhui Zhou, Hongbin Yuan, Yue Wang

**Affiliations:** ^1^ Department of Histology and Embryology, College of Basic Medicine, Shanghai, China; ^2^ Department of Anesthesiology, Changzheng Hospital, Shanghai, China; ^3^ NMPA Key Laboratory for Research and Evaluation of Narcotic and Psychotropic Drugs, Xuzhou Medical University, Xuzhou, China; ^4^ Department of Orthopedics, Chang Zheng Hospital, Shanghai, China

**Keywords:** mesenchymal stem cell–derived exosomes, hydrogel stiffness, sciatic nerve injury, nerve injury inflammation, exosome release behavior

## Abstract

A hydrogel system loaded with mesenchymal stem cell–derived exosome (MSC-Exos) is an attractive new tool for tissue regeneration. However, the effect of the stiffness of exosome-loaded hydrogels on tissue regeneration is unclear. Here, the role of exosome-loaded hydrogel stiffness, during the regeneration of injured nerves, was assessed *in vivo*. The results showed that the photocrosslinkable hyaluronic acid methacrylate hydrogel stiffness plays an important role in repairing nerve injury. Compared with the stiff hydrogels loaded with exosomes, soft hydrogels loaded with exosomes showed better repair of injured peripheral nerves. The soft hydrogel promoted nerve repair by quickly releasing exosomes to inhibit the infiltration of macrophages and the expression of the proinflammatory factors IL-1β and TNF-α in injured nerves. Our work revealed that exosome-loaded hydrogel stiffness plays an important role in tissue regeneration by regulating exosome release behavior and provided important clues for the clinical application of biological scaffold materials.

## Introduction

Exosomes, phospholipid bilayer vesicles with a diameter of 70–150 nm, have clinical application potential (Fais et al., 2016). Mesenchymal stem cell–derived exosomes (MSC-Exos) can promote the recovery of various injuries (Gyorgy et al., 2015; Malda et al., 2016). Previous research has shown that MSC-Exos can promote tissue regeneration and functional recovery during nerve injury (Sun et al., 2018), myocardial infarction (Yao et al., 2021), and liver transplantation (Lou et al., 2017). Therefore, MSC-Exos carrying mesenchymal stem cell (MSC)–related proteins, lipids, and nucleic acids are expected to replace MSCs as a safe cell-free clinical treatment tool (Malda et al., 2016; Cheng et al., 2017). Umbilical cord–derived mesenchymal stem cells are widely used in tissue engineering due to their easier collection (Weiss and Troyer, 2006; Howard et al., 2008). Porous hydrogels, as scaffolds to replace the extracellular matrix (ECM) of damaged tissues, are widely used in tissue repair (Prince and Kumacheva, 2019). Recently, studies have shown that porous hydrogels, as exosome delivery systems, can effectively prevent exosomes from being cleared by blood circulation and maintain an effective concentration of exosomes at the injury site (Riau et al., 2019; Huang et al., 2021). The hydrogel-loaded exosome system exhibits attractive clinical application potential for the skin (Henriques-Antunes et al., 2019; Shen et al., 2021), nerve (Li L. et al., 2020; Zhang et al., 2021a), bone (Liu et al., 2017; Jiang et al., 2021; Pishavar et al., 2021), and heart (Waters et al., 2018; Zhu et al., 2021) tissue repair. Studies have shown that hydrogel stiffness plays a key role in tissue damage repair (Prince and Kumacheva, 2019; Chaudhuri et al., 2020). However, the effect of exosome-loaded hydrogel stiffness on tissue damage repair is still unclear.

Peripheral nerve injury (PNI) is a common clinical disease that can be caused by blunt injury, penetrating injury, and crush damage. (Akar et al., 2021). PNI has been reported in 2–3% of trauma patients. Sensory, dyskinesia, or autonomic nerve dysfunction of the limbs caused by PNI severely affects patient quality of life (Luzhansky et al., 2019). After nerve injury, inflammation caused by the injury is the main factor causing nerve regeneration. Macrophages play a key role in the process of nerve injury and inflammation (Liu et al., 2019). The proinflammatory M1 subtype of macrophages promotes inflammation and increases tissue necrosis by releasing TNF-a and IL-1B (Liu et al., 2019). Although a variety of surgical and nonsurgical treatment methods have been approved for clinical use, the efficacy is unsatisfactory. Therefore, new treatments for PNI need to be developed urgently (Zhang et al., 2021b). As a classic model, rat sciatic nerve crush injury (SNCI) is widely used in the study of the repair process of PNI (Savastano et al., 2014; Akar et al., 2021). After surgery in mice with SNI, the motor and sensory disturbances caused by nerve damage were quantitatively evaluated through the sciatic functional index (SFI) and rat gastrocnemius morphological indicators (Seltzer et al., 1990).

Hence, photocrosslinkable hyaluronic acid methacrylate (HAMA) with different mechanical properties was selected to prepare hydrogels for loading of exosomes to repair sciatic nerve injury due to its good histocompatibility (Levett et al., 2014; Eke et al., 2017; Schuurmans et al., 2021). We used the rat SNCI model to determine the effect of exosome-loaded hydrogel stiffness on the repair of PNI. Our results showed that the stiffness of exosome-loaded hydrogels plays an important role in PNI and that the stiffness of loaded exosome hydrogels affects PNI repair by regulating the exosome release rate.

## Materials and Methods

### Cell Culture

Mycoplasma-negative human umbilical cord–derived mesenchymal stem cells (UMSCs) were cultured in low-sugar DMEM (11885084, Gibco) containing 10% fetal bovine serum (FBS, 10099141, Gibco), 100 U mL^−1^ penicillin, and 100 μg ml^−1^ streptomycin (15070063, Gibco). Mycoplasma-negative THP1 cells were cultured in RPMI-1640 (R2405, Sigma) containing 10% FBS (10099141, Gibco), 100 U mL^−1^ penicillin, and 100 μg ml^−1^ streptomycin (15070063, Gibco). All cells were cultured at 5% CO_2_ and 37°C.

### Collection of Supernatant Containing Exosomes

UMSCs were cultured to 70–80% confluence. After three washes with PBS (70011044, Gibco), the cells were cultured in a medium with exosome-free serum. The supernatant was collected after 48 h of incubation.

### Macrophage Induction

For M0 macrophages, 100 ng/ml phorbol 2-myristate 13-acetate (PMA, P8139, Sigma) was added to the THP1 medium, and THP1 cells began to adhere after 12 h of culture. For M1 macrophages, the PMA-induced THP1 cells were replaced with the medium, and then, 100 ng/ml lipopolysaccharide (LPS, SMB00610, Sigma) was added to the medium. After 24 h of induction, the total cell RNA was extracted for quantitative real-time PCR (qRT-PCR) (Ding et al., 2021).

### Exosome Collection and Characterization

UMSC exosomes were collected as described previously (Guo et al., 2019). Briefly, the collected supernatants were centrifuged at 1,500 × g for 20 min at 4°C to remove cell debris and then filtered through a 0.22-μm filter (SLGP033RB, Millipore). Next, the collected supernatants were centrifuged at 150,000 × g for 2 h at 4°C, and then the exosome pellet was resuspended in pure water or in 250 μl of RIPA lysis buffer (89901, Thermo) with Pierce protease inhibitor mini tablets (A32953, Thermo) for Western blotting.

### Characterization of the Number and Size of Exosomes

Exosome particle size and number were measured by Nanoflow (Apogee A50-micro, Apogee) according to the user manual. The exosome data were collected and analyzed by Apogee Histogram software.

### Transmission Electron Microscopy

The exosomes dissolved in PBS were aspirated, 10 μl was added dropwise to the copper mesh and allowed to settle for 1 min, and a filter paper was used to absorb the floating liquid. Ten microliters of phosphotungstic acid were added dropwise to the copper net to settle for 1 min, and the floating liquid was absorbed by the filter paper. The samples were dried for a few minutes at room temperature. Electron microscopy (HT-7700, Hitachi) imaging at 80 KV was performed.

### Exosome Quantification

The protein in the exosome solution, obtained by centrifugation, was quantified to measure the concentration of exosomes (Sun et al., 2018). The exosome protein concentration was characterized by BCA (23225, Thermo), and then, the concentration of the exosomal solution was adjusted to 1 μg/μl. Then, NanoFlow was used to characterize the number of exosome particles, and the results showed that the number of exosome particles was 1.1 × 10^8^ Evts/μg.

### Western Blot

Exosomal proteins were separated by polyacrylamide gel electrophoresis, and the proteins were transferred onto PVDF membranes (Millipore, IPVH00010) by wet transfer. The membranes were blocked with 5% (m/v) BSA in PBS for 3 h and then incubated with 1:500 CD9 (ab92726, Abcam) or 1:500 ALIX (ab186429, Abcam) primary antibodies overnight at 4°C. Horseradish peroxidase (HRP)–conjugated secondary antibody was incubated for 1 h at room temperature after washing the membranes with 0.1% Tween 20 in PBS. The protein blots were developed by a chemiluminescence system.

### Preparation and Characterization of Hydrogels With Different Stiffnesses

Photopolymerized HAMA, which was purchased from EFL (Suzhou Intelligent Manufacturing Research Institute), was used to prepare hydrogels with different mechanical properties according to the instructions. HAMA (150 kD) was dissolved in pure water containing 0.25% lithium phenyl (2,4,6-trimethylbenzoyl) phosphinate at concentrations of 200 μg/μl and 80 μg/μl. Then, different concentrations of HAMA solution were mixed with equal volumes of a solution of exosomes (1 μg/μl) or pure water (the final concentrations of HAMA were 100 μg/μl and 40 μg/μl, and the final concentration of exosomes was 0.5 μg/μl). Then, the HAMA solutions with different concentrations of added exosomes were cured by a 405-nm, 25-mW/cm^2^ light source for 30 s to obtain hydrogels with different mechanical properties. HAMA at different concentrations was photocrosslinked to form hydrogels. The mechanical properties were characterized using a rotational rheometer (HAAKE MARS III; Thermo) (Liu et al., 2020).

### Fluorescent Labeling With Exosomes

PKH26 (UR52302, Umibio) was used to label exosomes according to the instructions and previous descriptions (Xu et al., 2017). After fluorescent labeling, the exosomes were collected by ultracentrifugation. Then, the exosome pellet was resuspended in pure water. Nanoflow was used to characterize the number of PKH26-labeled exosome particles, and the final concentration was adjusted to 1.1 × 10^8^ Evts/µl (1 μg/μl).

### Experimental Animals

Male Sprague–Dawley (SD) rats weighing 100–200 g were provided by the Animal Experiments Center of Naval Medical University (Shanghai, China). All the rats were housed in standard rat cages at a temperature of 23°C and a humidity of 55% under a 12 h light–dark cycle. All the animal experiments were approved by the Scientific Investigation Committee of Naval Medical University (Animal ethics certificate number: 2017-0004) and followed the guidelines of the Ethics Committee of the International Pain Research Association (https://www.iasp-pain.org).

### SNCI Model and Experimental Protocol

The rat SNCI model was established according to the method described previously (Li R. et al., 2020). In brief, the rats were anesthetized with 4% pentobarbital sodium (30 mg/kg, i.p.), and the left sciatic nerve was exposed by separating the gluteal muscles. For the damage of the sciatic nerve axons, vascular forceps clamped the sciatic nerve for 40 s without damaging the nerve epineurium. The experimental rats were divided into the following groups. The same anesthesia and surgery were performed without damaging the sciatic nerve. For the control group, after SNCI, each rat received 100 μl of PBS after sciatic nerve damage. For the stiff group, after SNCI, 100 μl of HAMA solution (100 μg/μl) carrying exosomes (PKH26 labeled or unlabeled, 0.5 μg/μl) was dropped on the periphery of the injured nerve and photocrosslinked to form a hydrogel. For the soft group, after SNCI, 100 μl of HAMA solution (400 μg/μl) carrying exosomes (PKH26 labeled or unlabeled, 0.5 μg/μl) was dropped on the periphery of the injured nerve, and photocrosslinking was performed to form a hydrogel. For the stiff-hydrogel only group, after SNCI, 100 μl of HAMA solution (100 μg/μl) was dropped on the periphery of the injured nerve, and photocrosslinking was performed to form a hydrogel. For the soft-hydrogel only group, after SNCI, 100 μl of HAMA solution (40 μg/μl) was dropped on the periphery of the injured nerve, and photocrosslinking was performed to form a hydrogel.

### Fluorescence Imaging

Three-dimensional imaging of hydrogels loaded with PKH26-labeled exosomes: PKH26-labeled exosomes were loaded in a gel (1 μg/μl), and the hydrogel was 3D-imaged using a laser scanning confocal microscope (LSCM, SP8, Leica).

### Cellular Fluorescence Imaging

A total of 40 μl of HAMA solution at different concentrations (200 μg/μl or 80 μg/μl) was mixed with an equal volume of PKH26-labeled exosomes (1.1 × 10^8^ Evts/µl, 1 μg/μl), dropped on the bottom of a confocal dish, and photocrosslinked to form hydrogels with different mechanical properties. A total of 1 × 10^5^ THP1 cells were seeded on the hydrogel, 100 ng/ml PMA was added to the medium, the cells adhered to the wall after 12 h of induction, and the cells were fixed with paraformaldehyde after 24 h of culture and imaged with an LSCM. A total of 1 × 10^5^ THP1 cells were seeded in a confocal dish, and 100 ng/ml PMA was added to the culture medium to induce for 24 h. Then, 100 ng/ml LPS was added to the cells, and different concentrations of exosomes (10 μg/ml or 100 μg/ml) were added to the medium at the same time. After 24 h of culture, the cells were fixed and imaged with an LSCM. All LSCM images were collected using Leica Application Suite X software.

### Fluorescence Imaging of Tissue Sections

The sciatic nerve and dorsal root ganglion (DRG, L_4_) of the rats in the stiff and soft groups (loaded with PKH26-labeled exosomes) were removed 24 h after the operation to prepare the frozen sections. The frozen sections were blocked with 10% positive PBS for 1 h at room temperature and then stained with DAPI for 15 min. After mounting of the slide with 10 μl of antifluorescence attenuating agent, imaging was performed using a fluorescence microscope (Axio Observer, Zeiss). The sciatic nerves of the rats in the stiff and soft groups (loaded with unlabeled exosomes) were removed 3 days or 14 days after the operation to prepare the frozen sections. After antigen retrieval, the frozen sections were blocked with 5% BSA. CD68 (3 days, GB113109, Servicebio) or β-tubulin 3 (14 days, 5568T, CST) primary antibody at 1:200 was incubated overnight at 4°C. The cells were incubated for 3 h with a secondary antibody (GB22301, GB21303, and Servicebio), and the nuclei were stained with DAPI, mounted with an antifluorescence quencher and imaged with a fluorescence microscope. All the images were collected by ZEN2012 software.

### Function and Morphology Analysis of Rats With Sciatic Nerve Injury

SFI: After the rat’s hind feet were stained with red dye, they were placed in a track covered with white paper (60 cm long and 10 cm wide). The collected hind footprints were analyzed according to a previous description (Varejao et al., 2004). The SFI was calculated according to the following equation:

$$SFI=-38.3(\frac{EPL-NPL}{NPL})+109.5(\frac{ETS-NTS}{NTS})+13.3(\frac{EIT-NIT}{NIT})-8.8.$$ (1)

PL indicates the print length, and the value is the distance from the heel to the third toe. TS indicates the toe spread, and the value is the distance from the first to the fifth toe. IT indicates the intermediary toe spread, and the value is the distance from the second to the fourth toe. E represents the surgical side, and N represents the normal side. An SFI value of 0 indicates normal sciatic nerve function, and an SFI value of -100 indicates complete loss of sciatic nerve function.

### Gastrocnemius Wet Weight

The rat’s bilateral gastrocnemius muscles were removed and weighed immediately. The 100% value of the wet gastrocnemius muscle weight on the operating side/wet gastrocnemius muscle weight on the normal side was used to evaluate gastrocnemius atrophy caused by sciatic nerve damage (Singh et al., 2021). Moreover, the gastrocnemius muscle was cross-sectioned, and H&E staining analysis of the muscle fiber area was performed to further assess the level of muscle atrophy (Chen et al., 2020).

### H&E Staining and Immunohistochemical Staining

The injured sciatic nerve was wrapped with a hydrogel loaded with exosomes with different mechanical properties. The nerves and gastrocnemius muscle were removed 14 days later to prepare the paraffin sections. H&E staining: the rat gastrocnemius muscle was transected and stained with hematoxylin for 30 min at room temperature. The samples were rinsed with tap water for 15 min and soaked in hydrochloric acid. Then, the slices were dehydrated and stained with 0.5% erythritol for 3 min at room temperature. Then, they were rinsed with 95% alcohol. The samples were dehydrated with absolute ethanol, placed in xylene, and sealed with neutral gum, and a microscope (CX43; Olympus) was used to observe and image the samples.

### Immunohistochemical Staining

The rat sciatic nerve was cut longitudinally, and after antigen retrieval, the sections were blocked with 5% BSA. Then, 1:200 IL-1β (GB11113, Servicebio) or TNF-α (GB11188, Servicebio) primary antibody was incubated overnight at 4°C. The cells were incubated for 40 min with HRP-labeled secondary antibody (G1215, Servicebio). After AEC color development, hematoxylin was used to stain the nucleus. The image was observed under a microscope (CX43; Olympus) after mounting the slide.

### Q-RT-PCR

The total RNA of the cells was extracted using TRIzol (15596026, Invitrogen), and then, the RNA was reverse-transcribed for cDNA using a cDNA reverse transcription kit (4374967, Applied Biosystems). Finally, the SYBR Green ER (11762500, Invitrogen) mixture was used for the test on a QuantStudio 3 (Applied Biosystems). All data were collected and analyzed using QuantStudio Real-Time PCR software. The primer sequences are listed below. GAPDH: forward primer: ACA​ACT​TTG​GTA​TCG​TGG​AAG​G, reverse primer: GCC​ATC​ACG​CCA​CAG​TTT​C; TNFa: forward primer: GAG​GCC​AAG​CCC​TGG​TAT​G, reverse primer: CGG​GCC​GAT​TGA​TCT​CAG​C (PrimerBank ID, 25952110c2); and IL-1B: forward primer: ATG​ATG​GCT​TAT​TAC​AGT​GGC​AA, reverse primer: GTC​GGA​GAT​TCG​TAG​CTG​GA (PrimerBank ID, 27894305c1).

### Data Analysis

All fluorescence imaging was analyzed using ImageJ software, as described previously (Jensen, 2013). All data were analyzed and calculated by Origin 2018.

## Results

### Hydrogels With Different Stiffnesses Loaded With Exosomes for the Treatment of Peripheral Injuries

**FIGURE 1:**
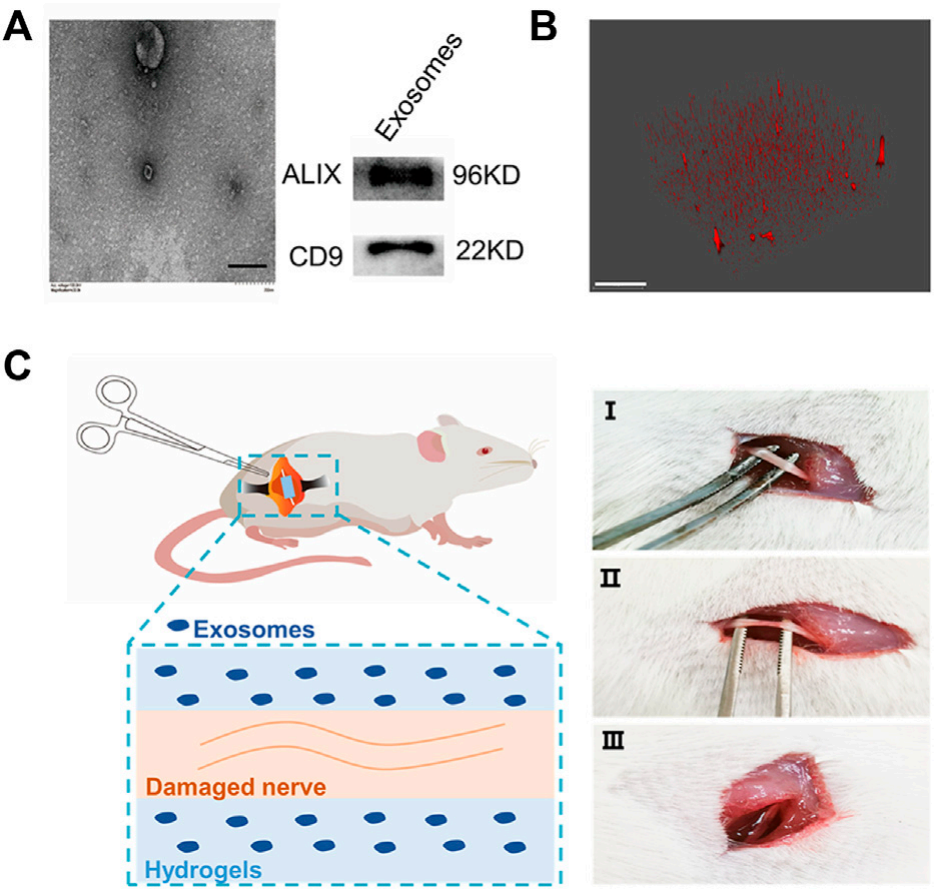
Hydrogel-loaded exosomes for the treatment of sciatic nerve injury. **(A)** Characterization of human umbilical cord–derived mesenchymal stem cell–derived exosomes (left: TEM of exosomes, bar = 200 nm; right: Western blot characterization of exosome marker proteins). **(B)** Fluorescence 3D imaging of hydrogels loaded with PKH26-labeled exosomes (red: PKH26, bar = 50 μm). **(C)** Rat SNCI model. Different concentrations of HAMA solution mixed with exosomes were injected into the injured nerve, and after curing with ultraviolet light, the hydrogels with different mechanical properties were formed at the nerve injury site (I, uninjured rat sciatic nerve; II, injured sciatic nerve; and III, injured nerve embedded in hydrogel).

Human umbilical cord–derived mesenchymal stem cell exosomes (uMSC-Exos) were collected by ultracentrifugation (Sun et al., 2018). Then, the morphological characteristics of exosomes were characterized by transmission electron microscopy (TEM) (Figure 1A, Supplementary Figure S4), and the size of the exosomes was characterized by NanoFlow (Supplementary Figure S1A). Furthermore, the exosome-specific protein markers (CD9 and ALIX) (Kowal et al., 2016; Jeppesen et al., 2019) were evaluated by Western blotting. The results showed that CD9-^−^ and ALIX-positive exosomes with complete morphology were collected. Then, HAMA hydrogels with different mechanical properties loaded with exosomes were prepared. The HAMA storage modulus was measured by using a rheometer (Supplementary Figure S1B); 0.78 KPa was defined as “soft” and 8.54 KPa was defined as “stiff.” To further characterize the distribution of exosomes in the hydrogel, we loaded PKH26-labeled exosomes into the hydrogel, and the 3D imaging results showed that the exosomes were distributed in the hydrogel (Li L. et al., 2020) (Figure 1B, Supplementary Figure S5). The abovementioned results indicated that MSC-Exos can be loaded into the HAMA hydrogel and distributed evenly. Then, the rat SNCI model was used to evaluate whether the stiffness of the exosome-loaded hydrogel affected nerve repair. The left sciatic nerve of the rat was exposed and crushed by forceps. One hundred microliters of different concentrations of HAMA with exosomes (0.55 × 10^8^ Evts/µl) was poured around the injured nerve, and the hydrogels with different mechanical properties were formed by photocrosslinking (Figure 1C).

### Stiffness of the Exosome-Loaded Hydrogels Affects Nerve Repair

**FIGURE 2:**
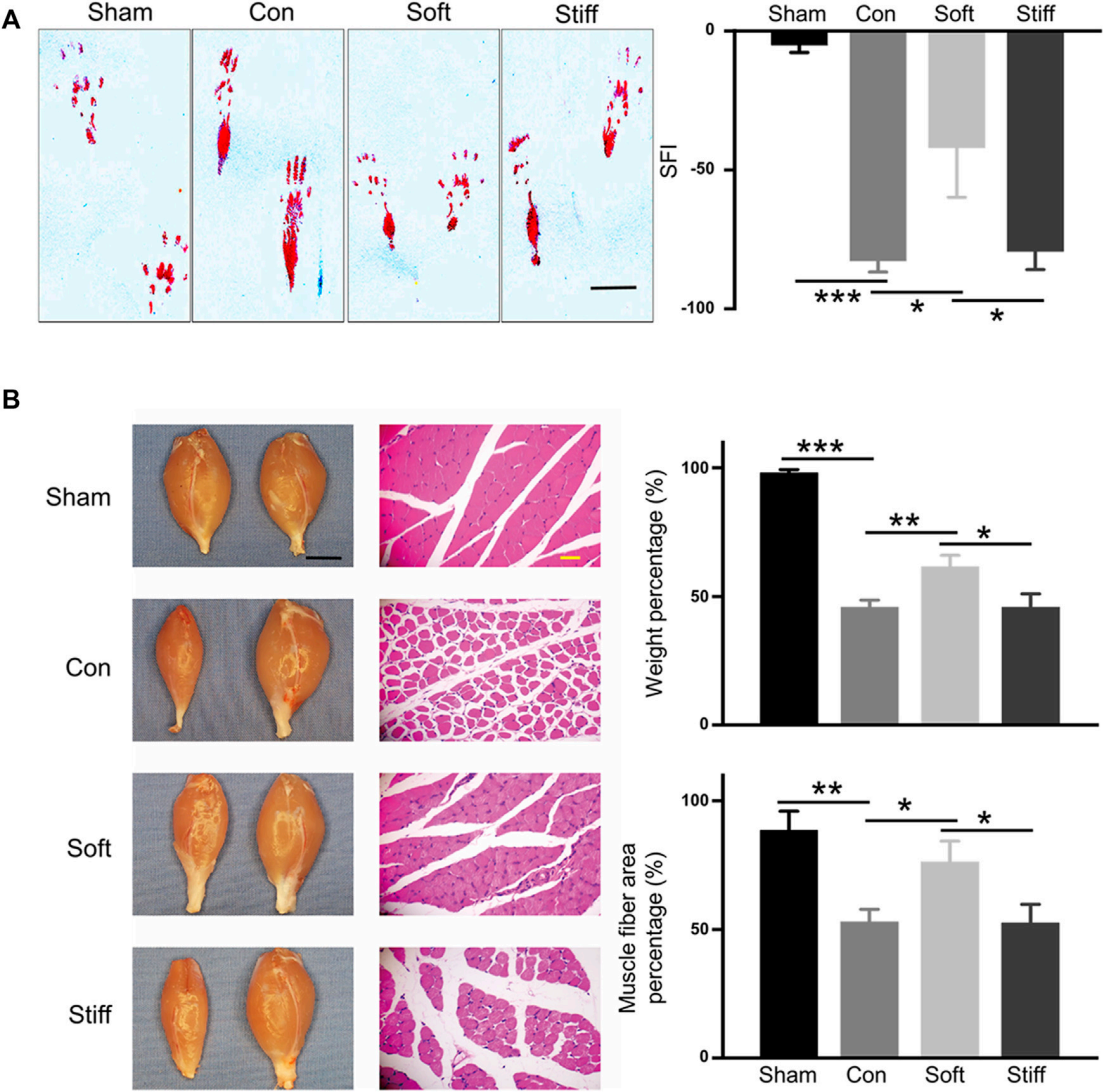
Soft hydrogel–loaded exosomes can better repair injured nerves. **(A)** Rat hind footprints were collected after surgery, and the SFI was used to evaluate the functional recovery of the injured sciatic nerve (left: typical footprint pattern, 7 days, bar = 1 cm; right: SFI score, 14 days). Con: surgery group, sham: sham operation group, con vs. sham *p* = 1.71649E-4; soft: soft hydrogel treatment group, soft vs. con *p* = 0.01813); and stiff: stiff hydrogel treatment group, soft vs. stiff *p* = 0.02697. **(B)** Wet weight of the gastrocnemius muscle and the cross-sectional area of the gastrocnemius muscle fibers. (left: photographs of rat gastrocnemius muscles on the operative and nonoperative sides and HE-stained images of the transverse section of the gastrocnemius muscle on the operating side, bar = 1 cm black, bar = 100 μm yellow).

The rats were divided into four groups: sham, in which only the sciatic nerve was exposed without SNCI; con, where 100 μl of PBS was dripped into the injured site after SNCI; stiff, the stiff hydrogel loaded with exosomes (0.55×10^8^ Evts/µl) was formed in the injured site after SNCI; and soft, soft hydrogel loaded with exosomes (0.55 × 10^8^ Evts/µl) was formed in the injured site after SNCI. Fourteen days after the operation, the rat’s SFI was assessed to evaluate the rat’s sciatic nerve function (Varejao et al., 2004; Chen et al., 2020; Lopez-Silva et al., 2021). The results showed that dysfunction caused by sciatic nerve injury significantly affected the rat git (Figure 2. A left) and significantly reduced the SFI (Figure 2. sham vs. con, from −5.05 to −82.70). The soft hydrogel loaded with exosomes significantly increased the SFI value of the rats after sciatic nerve injury (Figure 2. A soft vs. con, from -82.70 to -47.88), and it resulted in a greater increase in the SFI value than that of the stiff (−79.36) hydrogel loaded with exosomes (Figure 2. A soft vs. stiff). The gait changes of the rats are accompanied by atrophy of the gastrocnemius muscle, so the wet weight and muscle fiber area of the gastrocnemius of different groups of rats were also analyzed (Chen et al., 2020; Lopez-Silva et al., 2021; Singh et al., 2021). The results showed that sciatic nerve injury resulted in atrophy of the gastrocnemius muscle on the operating side, a decrease in wet weight, and a decrease in muscle fiber area (Figure 2. B sham vs. con; weight from 98.04% to 45.81%; area from 88.55% to 53.01%). The soft hydrogel loaded with exosomes had a significant therapeutic effect on gastrocnemius atrophy after sciatic nerve injury (Figure 2. B soft vs. con; weight increased from 45.81% to 61.63%; area increased from 53.01% to 76.34%), and the therapeutic effect was significantly better than that of the stiff (weight 45.85%, area 52.56%) hydrogel loaded with exosomes (Figure 2. B soft vs. stiff).

Previous studies have shown that the mechanical properties of biomaterials affect damage repair (Kratochvil et al., 2019; Chaudhuri et al., 2020; Khare et al., 2021). Therefore, the repair effects of hydrogels with different stiffnesses without exosomes were evaluated. The rats were divided into two groups: stiff-hydrogel only, in which the stiff hydrogel was formed in the injured site after SNCI; and the soft-hydrogel only, in which the soft hydrogel was formed in the injured site after SNCI. Fourteen days after the operation, the wet weight and muscle fiber area of the rat gastrocnemius muscle were analyzed. The results showed that there was no significant difference between the soft hydrogel and the stiff hydrogel in repairing muscle atrophy caused by sciatic nerve injury without exosomes (Supplementary Figure S2). The abovementioned results indicated that the exosome behavior regulated by hydrogel stiffness affects the repair effect of sciatic nerve injury.

### The Exosome-Loaded Soft Hydrogel Promotes the Repair of Injured Nerves by Inhibiting the Inflammatory Response

**FIGURE 3:**
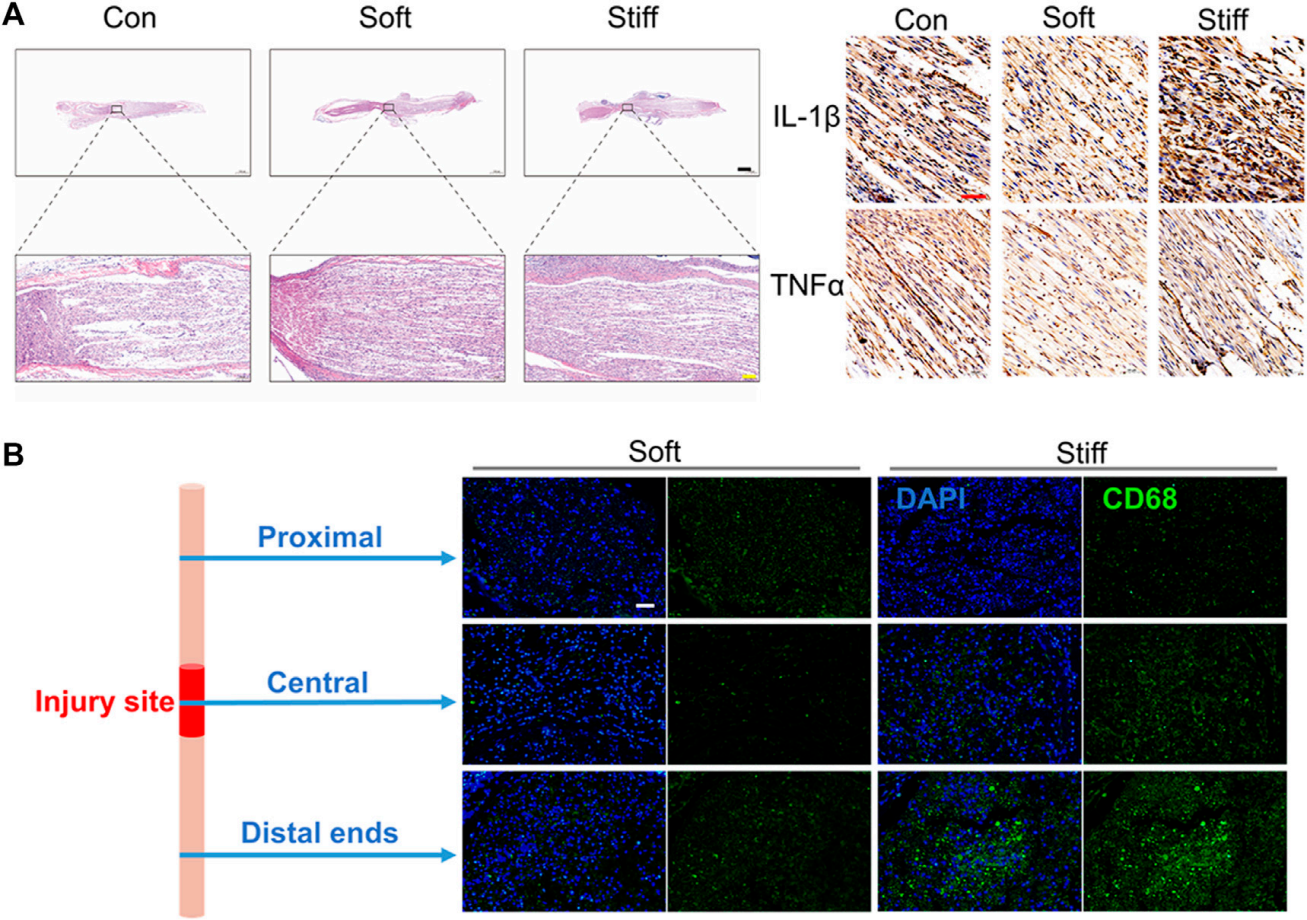
Soft hydrogels loaded with exosomes better repair nerve damage by inhibiting inflammation. **(A)** H&E staining imaging of the injured nerves (left: 14 days, bar = 1,000 μm black, bar = 100 μm yellow). Immunohistochemistry imaging of IL-1β and TNF-α (right: 14 days, bar = 50 μm red). **(B)** Immunofluorescence imaging of the injured nerves (1 day, bar = 50 μm, green: CD68, blue: DAPI).

Fourteen days after the operation, the HE staining results of the injured sciatic nerve showed that compared with the stiff group, the soft group showed better repair of the injured nerves, displaying a more regular arrangement (Figure 3A left). Injury-induced inflammation is an important factor that hinders the repair of sciatic nerve injury (Liu et al., 2019). Previous studies have proven that uMSC-Exos can significantly inhibit inflammation caused by sciatic nerve injury (Sun et al., 2018). Therefore, we hypothesized that the exosome-loaded soft hydrogels can inhibit the inflammation of injured nerves better than stiff hydrogels. Therefore, the levels of IL-1β and TNF-α in the injured nerves were evaluated by immunohistochemistry because the proinflammatory factors IL-1β and TNF-α, secreted by M1 macrophages after nerve injury, are important factors hindering nerve repair (Liu et al., 2019). The results showed that the levels of IL-1β and TNF-α in the damaged sciatic nerve were lower in the soft group than in the stiff group (Figure 3A right). We further evaluated the inhibitory effects of the exosome-loaded hydrogels with different stiffnesses on injured nerve inflammation at 24 h after the operation. Twenty-four hours after the operation, the injured sciatic nerves of the rats in the stiff group and the soft group were removed, and the macrophages were characterized by CD68 after freezing the sections (Lee et al., 2020; Lu et al., 2020). The results showed that the soft hydrogel loaded with exosomes can significantly inhibit the infiltration of macrophages on the nerve in the early stage of nerve injury, especially in the distal ends of the injured nerve (Figure 3B). In conclusion, the soft hydrogel–regulated exosome behavior promotes peripheral nerve repair by inhibiting the injury-induced inflammatory response. The soft hydrogel loaded with exosomes promotes nerve regeneration by inhibiting inflammatory response, thereby improving the SFI and increasing muscle mass and muscle fiber area.

### The Rapid Release of Exosomes Mediated by Soft Hydrogels Promotes the Repair of Injured Nerves by Inhibiting Inflammation

**FIGURE 4:**
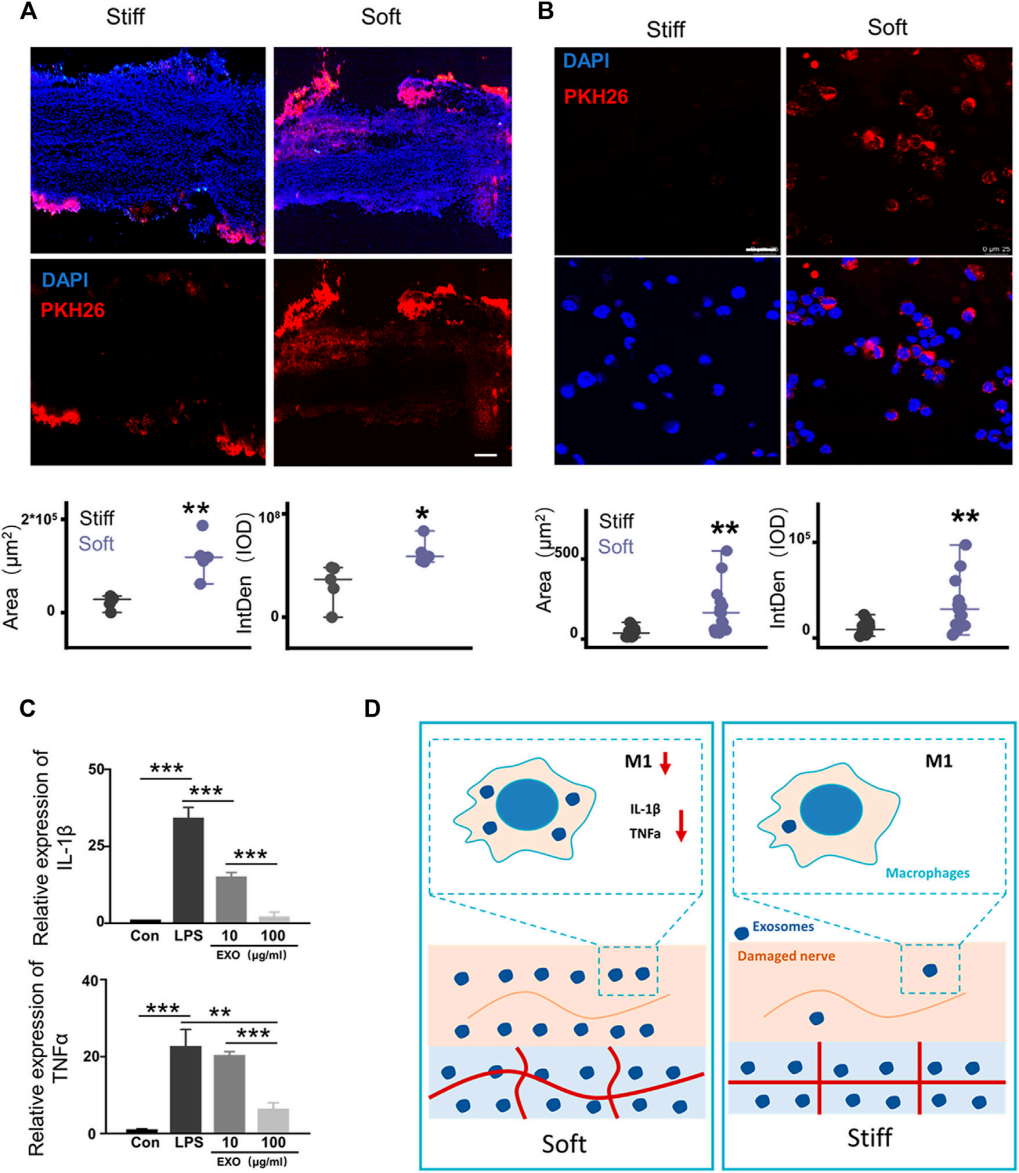
Rapid release of exosomes mediated by soft hydrogels inhibits macrophage inflammation. **(A)** After 24 h, the hydrogel-embedded sciatic nerves were collected, and the frozen sections were imaged under a fluorescence microscope. Image analysis showed that compared with the stiff hydrogel–embedded sciatic nerves, the sciatic nerves embedded in the soft hydrogels had a larger fluorescent area (*p* = 0.00162, n = 5) and higher IOD (*p* = 0.01827, n = 5; bar = 200 μm). **(B)** Hydrogels with different stiffnesses were loaded with PKH26-labeled exosomes and incubated with THP1 cells and PMA (100 ng/ml). After 24 h, the cells were imaged under a laser confocal microscope (bar = 250 μm). Image analysis shows that compared with the cells grown on stiff hydrogels, the cells grown on soft hydrogels had a larger fluorescent area (*p* = 0.00101, n = 15) and higher IOD (*p* = 0.00201, n = 15). **(C)** After PMA (100 ng/ml) induced THP1 to adhere to the wall (con), LPS (100 ng/ml) was used to induce the cells to differentiate into M1 for 12 h (LPS). Then, the cells were treated with different concentrations of exosomes to detect the expression of the related genes, IL-1β and TNF-α (IL-1β: con vs. LPS *p* = 6.57496E-5, LPS vs. 10 μg/ml *p* = 7.66959E-4, 10 μg/ml vs. 100 μg/ml *p* = 3.2624E-4; TNF-α: con vs. LPS *p* = 9.737936E-4, LPS vs. 10 μg/ml *p* = 0.00355, 10 μg/ml vs. 100 μg/ml *p* = 1.63632E-4; n = 3). **(D)** Schematic diagram of the mechanism by which exosome release affects damaged nerve repair. The rapid release of exosomes from the soft hydrogel can reduce macrophages and the expression of proinflammatory IL-1β and TNF-α in M1 macrophages, thereby promoting the repair of the injured nerve. *p < 0.05; **p < 0.01; and ***p < 0.001, *t* test.

Hydrogel stiffness regulates the diffusion of exosomes (Lenzini et al., 2020). Therefore, we hypothesized that soft hydrogels promote neural repair by modulating exosome release. The rat SNCI model was used to evaluate the regulation of hydrogel stiffness on the release of exosomes in the *in vivo* microenvironment. The left sciatic nerve of the rat was exposed and crushed by forceps. One hundred microliters of different concentrations of HAMA with PKH26-labeled exosomes (0.55 × 10^8^ Evts/µl) were dripped around the injured nerve, and hydrogels with different mechanical properties were formed by photocrosslinking (Figure 4A). Twenty-four hours after the surgery, the rat sciatic nerve was removed, frozen, sectioned, and then fluorescently imaged. The results showed that compared with that in the stiff hydrogel, the sciatic nerve wrapped in the soft hydrogel has a larger fluorescent area and higher integrated optical density (Figure 4A).

The hydrogel, as a substitute for the ECM, forms cell–hydrogel interfaces with the tissue cells of the injury when repairing the tissues (Stevens and George, 2005; Li et al., 2019). Therefore, the effects of hydrogels with different stiffnesses on regulating exosome release in the cell–hydrogel interface system were evaluated. One hundred microliters of different concentrations of HAMA with PKH26-labeled exosomes (0.55 × 10^8^ Evts/µl) was dropped at the bottom of the confocal dish, and the hydrogels with different mechanical properties were formed by photocrosslinking. A total of 1 × 10^5^ THP1 cells were seeded on the hydrogel with 100 ng/ml PMA (Ding et al., 2021). After PMA induction for 12 h, the cells adhered to the hydrogel with different stiffnesses. After 24 h, the cells were fluorescently imaged. Fluorescence imaging showed that compared with the cells grown on the stiff hydrogels, the cells grown on the soft hydrogels had a larger fluorescent area and higher integrated optical density (IOD, Figure 4B). The soft hydrogels released exosomes more rapidly in the cell–hydrogel interface system than the stiff hydrogels after 24 h. The abovementioned results showed that the dose difference of exosomes formed at the injured peripheral nerve site is due to hydrogel stiffness, modulating exosome release. Therefore, we used different concentrations of exosomes to treat the THP1 cells that were M2-polarized by LPS induction, and the results showed that higher concentrations of exosomes can better inhibit the expression of IL-1β and TNF-α in M1 macrophages (Figure 4C). Our results showed that compared with the stiff hydrogels, the soft hydrogels that rapidly release exosomes can better repair PNI and motor dysfunction by reducing the infiltration of macrophages into the injured nerve in the early stage of nerve injury. This phenomenon reduces the number of M1 macrophages in the injured nerve. The rapid release of high doses formed by exosomes can better inhibit the expression of the proinflammatory factors IL-1β and TNF-α in macrophages (Figure 4D). Compared with the stiff hydrogels that release exosomes slowly, the soft hydrogels that quickly release exosomes can better repair the injured sciatic nerves by inhibiting inflammation.

## Discussion

The mechanical properties of biomaterials play an important role in tissue repair (Kratochvil et al., 2019; Chaudhuri et al., 2020; Khare et al., 2021). However, the role of exosome-loaded hydrogel scaffold stiffness in tissue repair is still unclear. Our work showed that the stiffness of the exosome-loaded hydrogel affects neural repair. Compared with the stiff hydrogels, the soft hydrogels can promote nerve repair by inhibiting the inflammatory response caused by injury. Hydrogel mechanical properties regulate exosome diffusion behavior (Lenzini et al., 2020). Therefore, we focused on the differences in the diffusion behavior of the exosomes in hydrogels with different stiffness *in vivo*. Further analysis found that the soft hydrogel inhibits the inflammatory response caused by injury by rapidly releasing exosomes.

Our work revealed an opposite result to that of the previous work (Lenzini et al., 2020). Stiff hydrogels release exosomes faster in the previous work, while the soft hydrogels release exosomes faster in our results. Since the data of the previous work were derived from *in vitro* experiments, therefore we further evaluated the diffusion behavior of exosomes loaded in the hydrogels with different stiffnesses *in vitro*. 100 μl of different concentrations of HAMA with exosomes (0.55 × 10^8^ Evts/µl) was added to the 48-well plate, and hydrogels with different mechanical properties were formed by photocrosslinking. Then, 200 μl of PBS was added dropwise to the wells to cover the hydrogel. PBS was collected from the wells every 24 h, fresh PBS was added, and the number of exosome particles in PBS was measured by Nanoflow. Compared with the soft hydrogel, the stiff hydrogel released more exosomes into the solution after 24 h (Supplementary Figure S3). However, after 48 h, there was no longer a difference in the release rate of exosomes from hydrogels with different mechanical properties (Supplementary Figure S3). The abovementioned results showed an interesting contradictory trend of the exosome release rate in the solution system and the cell–hydrogel interface system regulated by hydrogel stiffness. In the solution system, the stiff hydrogel releases exosomes more quickly, while the soft hydrogel releases exosomes more efficiently in the cell-hydraulic interface system. The abovementioned results demonstrate that our results, contrary to those of previous work (Lenzini et al., 2020), are due to the *in vivo* microenvironment. There are usually differences in the release kinetics of exosomes from the scaffolds *in vivo* and *in vitro* (Huang et al., 2021). The abovementioned results showed the complexity of the release kinetics of exosomes from the scaffolds in the *in vivo* microenvironment, and the release kinetics will be systematically discussed in our follow-up work.

Exosome release behaviors affect tissue repair (Henriques-Antunes et al., 2019). We examined the regulation of the exosome release rate by hydrogel stiffness and analyzed the effect of exosome release behavior regulated by hydrogel stiffness on the repair of PNI. Injury inflammation is an important reason that hinders the repair of sciatic nerve injury (Liu et al., 2019). Previous studies have proved that uMSC-Exo can significantly inhibit inflammation (Sun et al., 2018), and our data show the dose effect of exosomes on the inhibition of M1 macrophage–mediated inflammation (Figure 4C). Our results show that the soft hydrogels promote nerve regeneration through the rapid release of exosomes resulting in high-dose effects at the early stage of nerve injury. However, the mechanism by which the exosome-loaded hydrogels inhibit inflammation needs to be further analyzed. The abovementioned results provide important clues for the design of exosome scaffolds for tissue injury repair.

## Conclusion

In summary, we investigated the role of exosome-loaded hydrogel stiffness in nerve injury repair *in vivo.* Compared with that of the stiff hydrogels, the rapid release of exosomes regulated by soft hydrogels can better repair PNI and motor dysfunction *in vivo*. The stiffness of exosome-loaded hydrogels affects PNI repair by modulating the rate of exosome release. Compared with stiff hydrogels, soft hydrogels promote nerve regeneration by rapidly releasing exosomes to suppress the inflammatory response caused by PNI. Our work has revealed that exosome release controlled by hydrogel stiffness plays an important role in tissue repair and provides important clues for the clinical application of hydrogels.

## Data Availability

The datasets presented in this study can be found in online repositories. The names of the repository/repositories and accession number(s) can be found in the article/Supplementary Material.

## References

[B1] Akar E. Tural Emon S. Tural Emon S. Efendioglu M. Erdogan B. Engin T. (2021). Effects of Tamoxifen Therapy on Sciatic Nerve Crush Injury: An Experimental Study in Rats. Aott 55 (2), 87–93. 10.5152/j.aott.2021.19183 33847568 PMC11229617

[B2] Chang F. Wang Y. Liu P. Peng J. Han G.-H. Ding X. (2019). Role of Macrophages in Peripheral Nerve Injury and Repair. Neural Regen. Res. 14 (8), 1335–1342. 10.4103/1673-5374.253510 30964051 PMC6524518

[B3] Chaudhuri O. Cooper-White J. Janmey P. A. Mooney D. J. Shenoy V. B. (2020). Effects of Extracellular Matrix Viscoelasticity on Cellular Behaviour. Nature 584 (7822), 535–546. 10.1038/s41586-020-2612-2 32848221 PMC7676152

[B4] Chen T. Li Y. Ni W. Tang B. Wei Y. Li J. (2020). Human Neural Stem Cell-Conditioned Medium Inhibits Inflammation in Macrophages via Sirt-1 Signaling Pathway *In Vitro* and Promotes Sciatic Nerve Injury Recovery in Rats. Stem Cells Dev. 29 (16), 1084–1095. 10.1089/scd.2020.0020 32560594

[B5] Cheng L. Zhang K. Wu S. Cui M. Xu T. (2017). Focus on Mesenchymal Stem Cell-Derived Exosomes: Opportunities and Challenges in Cell-free Therapy. Stem Cells Int. 2017, 1–10. 10.1155/2017/6305295 PMC5749272 29410682

[B6] Cheng Y.-B. Ren C. Geng D.-Q. Zhang R.-C. Du W.-Q. Zhang J.-Y. (2021b). Mesenchymal Stem Cell Treatment for Peripheral Nerve Injury: a Narrative Review. Neural Regen. Res. 16 (11), 2170–2176. 10.4103/1673-5374.310941 33818489 PMC8354135

[B7] Ding J. Zhang Y. Cai X. Zhang Y. Yan S. Wang J. (2021). Extracellular Vesicles Derived from M1 Macrophages Deliver miR-146a-5p and miR-146b-5p to Suppress Trophoblast Migration and Invasion by Targeting TRAF6 in Recurrent Spontaneous Abortion. Theranostics 11 (12), 5813–5830. 10.7150/thno.58731 33897883 PMC8058722

[B8] Eke G. Mangir N. Hasirci N. MacNeil S. Hasirci V. (2017). Development of a UV Crosslinked Biodegradable Hydrogel Containing Adipose Derived Stem Cells to Promote Vascularization for Skin Wounds and Tissue Engineering. Biomaterials 129, 188–198. 10.1016/j.biomaterials.2017.03.021 28343005

[B9] Fais S. O’Driscoll L. Borras F. E. Buzas E. Camussi G. Cappello F. (2016). Evidence-Based Clinical Use of Nanoscale Extracellular Vesicles in Nanomedicine. ACS Nano 10 (4), 3886–3899. 10.1021/acsnano.5b08015 26978483

[B10] Guo S. Perets N. Betzer O. Ben-Shaul S. Sheinin A. Michaelevski I. (2019). Intranasal Delivery of Mesenchymal Stem Cell Derived Exosomes Loaded with Phosphatase and Tensin Homolog siRNA Repairs Complete Spinal Cord Injury. ACS Nano 13 (9), 10015–10028. 10.1021/acsnano.9b01892 31454225

[B11] György B. Hung M. E. Breakefield X. O. Leonard J. N. (2015). Therapeutic Applications of Extracellular Vesicles: Clinical Promise and Open Questions. Annu. Rev. Pharmacol. Toxicol. 55, 439–464. 10.1146/annurev-pharmtox-010814-124630 25292428 PMC4445965

[B12] Henriques-Antunes H. Cardoso R. M. S. Zonari A. Correia J. Leal E. C. Jiménez-Balsa A. (2019). The Kinetics of Small Extracellular Vesicle Delivery Impacts Skin Tissue Regeneration. ACS Nano 13 (8), 8694–8707. 10.1021/acsnano.9b00376 31390518

[B13] Howard D. Buttery L. D. Shakesheff K. M. Roberts S. J. (2008). Tissue Engineering: Strategies, Stem Cells and Scaffolds. J. Anat. 213 (1), 66–72. 10.1111/j.1469-7580.2008.00878.x 18422523 PMC2475566

[B14] Huang J. Xiong J. Yang L. Zhang J. Sun S. Liang Y. (2021). Cell-free Exosome-Laden Scaffolds for Tissue Repair. Nanoscale 13 (19), 8740–8750. 10.1039/d1nr01314a 33969373

[B15] Jensen E. C. (2013). Quantitative Analysis of Histological Staining and Fluorescence Using ImageJ. Anat. Rec. 296 (3), 378–381. 10.1002/ar.22641 23382140

[B16] Jeppesen D. K. Fenix A. M. Franklin J. L. Higginbotham J. N. Zhang Q. Zimmerman L. J. (2019). Reassessment of Exosome Composition. Cell 177 (2), 428–445. 10.1016/j.cell.2019.02.029 30951670 PMC6664447

[B17] Jiang S. Tian G. Yang Z. Gao X. Wang F. Li J. (2021). Enhancement of Acellular Cartilage Matrix Scaffold by Wharton's Jelly Mesenchymal Stem Cell-Derived Exosomes to Promote Osteochondral Regeneration. Bioact. Mater. 6 (9), 2711–2728. 10.1016/j.bioactmat.2021.01.031 33665503 PMC7895679

[B18] Khare E. Holten-Andersen N. Buehler M. J. (2021). Transition-metal Coordinate Bonds for Bioinspired Macromolecules with Tunable Mechanical Properties. Nat. Rev. Mater 6 (5), 421–436. 10.1038/s41578-020-00270-z

[B19] Kowal J. Arras G. Colombo M. Jouve M. Morath J. P. Primdal-Bengtson B. (2016). Proteomic Comparison Defines Novel Markers to Characterize Heterogeneous Populations of Extracellular Vesicle Subtypes. Proc. Natl. Acad. Sci. U.S.A. 113 (8), E968–E977. 10.1073/pnas.1521230113 26858453 PMC4776515

[B20] Kratochvil M. J. Seymour A. J. Li T. L. Paşca S. P. Kuo C. J. Heilshorn S. C. (2019). Engineered Materials for Organoid Systems. Nat. Rev. Mater 4 (9), 606–622. 10.1038/s41578-019-0129-9 33552558 PMC7864216

[B21] Lee H.-G. Choi J.-H. Jang Y.-S. Kim U.-K. Kim G.-C. Hwang D.-S. (2020). Non-thermal Plasma Accelerates the Healing Process of Peripheral Nerve Crush Injury in Rats. Int. J. Med. Sci. 17 (8), 1112–1120. 10.7150/ijms.44041 32410841 PMC7211154

[B22] Lenzini S. Bargi R. Chung G. Shin J.-W. (2020). Matrix Mechanics and Water Permeation Regulate Extracellular Vesicle Transport. Nat. Nanotechnol. 15 (3), 217–223. 10.1038/s41565-020-0636-2 32066904 PMC7075670

[B23] Levett P. A. Melchels F. P. W. Schrobback K. Hutmacher D. W. Malda J. Klein T. J. (2014). A Biomimetic Extracellular Matrix for Cartilage Tissue Engineering Centered on Photocurable Gelatin, Hyaluronic Acid and Chondroitin Sulfate. Acta Biomater. 10 (1), 214–223. 10.1016/j.actbio.2013.10.005 24140603

[B24] Li C. Guo C. Fitzpatrick V. Ibrahim A. Zwierstra M. J. Hanna P. (2019). Design of Biodegradable, Implantable Devices towards Clinical Translation. Nat. Rev. Mater 5 (1), 61–81. 10.1038/s41578-019-0150-z

[B25] Li L. Zhang Y. Mu J. Chen J. Zhang C. Cao H. (2020a). Transplantation of Human Mesenchymal Stem-Cell-Derived Exosomes Immobilized in an Adhesive Hydrogel for Effective Treatment of Spinal Cord Injury. Nano Lett. 20 (6), 4298–4305. 10.1021/acs.nanolett.0c00929 32379461

[B26] Li R. Li D. Wu C. Ye L. Wu Y. Yuan Y. (2020b). Nerve Growth Factor Activates Autophagy in Schwann Cells to Enhance Myelin Debris Clearance and to Expedite Nerve Regeneration. Theranostics 10 (4), 1649–1677. 10.7150/thno.40919 32042328 PMC6993217

[B27] Liu X. Yang Y. Li Y. Niu X. Zhao B. Wang Y. (2017). Integration of Stem Cell-Derived Exosomes with *In Situ* Hydrogel Glue as a Promising Tissue Patch for Articular Cartilage Regeneration. Nanoscale 9 (13), 4430–4438. 10.1039/c7nr00352h 28300264

[B28] Liu Z. Wang L. Xu H. Du Q. Li L. Wang L. (2020). Heterogeneous Responses to Mechanical Force of Prostate Cancer Cells Inducing Different Metastasis Patterns. Adv. Sci. 7 (15), 1903583. 10.1002/advs.201903583 PMC7404165 32775149

[B29] Lopez-Silva T. L. Cristobal C. D. Edwin Lai C. S. Leyva-Aranda V. Lee H. K. Hartgerink J. D. (2021). Self-assembling Multidomain Peptide Hydrogels Accelerate Peripheral Nerve Regeneration after Crush Injury. Biomaterials 265, 120401. 10.1016/j.biomaterials.2020.120401 33002786 PMC7669633

[B30] Lou G. Chen Z. Zheng M. Liu Y. (2017). Mesenchymal Stem Cell-Derived Exosomes as a New Therapeutic Strategy for Liver Diseases. Exp. Mol. Med. 49 (6), e346. 10.1038/emm.2017.63 28620221 PMC5519012

[B31] Lu C.-Y. Santosa K. B. Jablonka-Shariff A. Vannucci B. Fuchs A. Turnbull I. (2020). Macrophage-Derived Vascular Endothelial Growth Factor-A Is Integral to Neuromuscular Junction Reinnervation after Nerve Injury. J. Neurosci. 40 (50), 9602–9616. 10.1523/JNEUROSCI.1736-20.2020 33158964 PMC7726545

[B32] Luzhansky I. D. Sudlow L. C. Brogan D. M. Wood M. D. Berezin M. Y. (2019). Imaging in the Repair of Peripheral Nerve Injury. Nanomedicine 14 (20), 2659–2677. 10.2217/nnm-2019-0115 31612779 PMC6886568

[B33] Malda J. Boere J. van de Lest C. H. A. van Weeren P. R. Wauben M. H. M. (2016). Extracellular Vesicles - New Tool for Joint Repair and Regeneration. Nat. Rev. Rheumatol. 12 (4), 243–249. 10.1038/nrrheum.2015.170 26729461 PMC7116208

[B34] Pishavar E. Luo H. Naserifar M. Hashemi M. Toosi S. Atala A. (2021). Advanced Hydrogels as Exosome Delivery Systems for Osteogenic Differentiation of MSCs: Application in Bone Regeneration. Ijms 22 (12), 6203. 10.3390/ijms22126203 34201385 PMC8228022

[B35] Prince E. Kumacheva E. (2019). Design and Applications of Man-Made Biomimetic Fibrillar Hydrogels. Nat. Rev. Mater 4 (2), 99–115. 10.1038/s41578-018-0077-9

[B36] Riau A. K. Ong H. S. Yam G. H. F. Mehta J. S. (2019). Sustained Delivery System for Stem Cell-Derived Exosomes. Front. Pharmacol. 10, 1368. 10.3389/fphar.2019.01368 31798457 PMC6868085

[B37] Savastano L. E. Laurito S. R. Fitt M. R. Rasmussen J. A. Gonzalez Polo V. Patterson S. I. (2014). Sciatic Nerve Injury: a Simple and Subtle Model for Investigating Many Aspects of Nervous System Damage and Recovery. J. Neurosci. Methods 227, 166–180. 10.1016/j.jneumeth.2014.01.020 24487015

[B38] Schuurmans C. C. L. Mihajlovic M. Hiemstra C. Ito K. Hennink W. E. Vermonden T. (2021). Hyaluronic Acid and Chondroitin Sulfate (Meth)acrylate-based Hydrogels for Tissue Engineering: Synthesis, Characteristics and Pre-clinical Evaluation. Biomaterials 268, 120602. 10.1016/j.biomaterials.2020.120602 33360302

[B39] Seltzer Z. e. Dubner R. Shir Y. (1990). A Novel Behavioral Model of Neuropathic Pain Disorders Produced in Rats by Partial Sciatic Nerve Injury. Pain 43 (2), 205–218. 10.1016/0304-3959(90)91074-s 1982347

[B40] Shen Y. Xu G. Huang H. Wang K. Wang H. Lang M. (2021). Sequential Release of Small Extracellular Vesicles from Bilayered Thiolated Alginate/Polyethylene Glycol Diacrylate Hydrogels for Scarless Wound Healing. ACS Nano 15 (4), 6352–6368. 10.1021/acsnano.0c07714 33723994

[B41] Singh A. Raghav A. Shiekh P. A. Kumar A. (2021). Transplantation of Engineered Exosomes Derived from Bone Marrow Mesenchymal Stromal Cells Ameliorate Diabetic Peripheral Neuropathy under Electrical Stimulation. Bioact. Mater. 6 (8), 2231–2249. 10.1016/j.bioactmat.2021.01.008 33553812 PMC7829156

[B42] Stevens M. M. George J. H. (2005). Exploring and Engineering the Cell Surface Interface. Science 310 (5751), 1135–1138. 10.1126/science.1106587 16293749

[B43] Sun G. Li G. Li D. Huang W. Zhang R. Zhang H. (2018). hucMSC Derived Exosomes Promote Functional Recovery in Spinal Cord Injury Mice via Attenuating Inflammation. Mater. Sci. Eng. C 89, 194–204. 10.1016/j.msec.2018.04.006 29752089

[B44] Varejão A. S. P. Cabrita A. M. Meek M. F. Bulas-Cruz J. Melo-Pinto P. Raimondo S. (2004). Functional and Morphological Assessment of a Standardized Rat Sciatic Nerve Crush Injury with a Non-serrated Clamp. J. Neurotrauma 21 (11), 1652–1670. 10.1089/neu.2004.21.1652 15684656

[B45] Waters R. Alam P. Pacelli S. Chakravarti A. R. Ahmed R. P. H. Paul A. (2018). Stem Cell-Inspired Secretome-Rich Injectable Hydrogel to Repair Injured Cardiac Tissue. Acta Biomater. 69, 95–106. 10.1016/j.actbio.2017.12.025 29281806 PMC5831493

[B46] Weiss M. L. Troyer D. L. (2006). Stem Cells in the Umbilical Cord. Stem Cell Rev. 2 (2), 155–162. 10.1007/s12015-006-0022-y 17237554 PMC3753204

[B47] Xu B. Zhang Y. Du X.-F. Li J. Zi H.-X. Bu J.-W. (2017). Neurons Secrete miR-132-Containing Exosomes to Regulate Brain Vascular Integrity. Cell Res. 27 (7), 882–897. 10.1038/cr.2017.62 28429770 PMC5518987

[B48] Yao J. Huang K. Zhu D. Chen T. Jiang Y. Zhang J. (2021). A Minimally Invasive Exosome Spray Repairs Heart after Myocardial Infarction. ACS Nano 15, 11099–11111. 10.1021/acsnano.1c00628 34152126

[B49] Zhang L. Fan C. Hao W. Zhuang Y. Liu X. Zhao Y. (2021a). NSCs Migration Promoted and Drug Delivered Exosomes‐Collagen Scaffold via a Bio‐Specific Peptide for One‐Step Spinal Cord Injury Repair. Adv. Healthc. Mat. 10 (8), 2001896. 10.1002/adhm.202001896 33522126

[B50] Zhu D. Li Z. Huang K. Caranasos T. G. Rossi J. S. Cheng K. (2021). Minimally Invasive Delivery of Therapeutic Agents by Hydrogel Injection into the Pericardial Cavity for Cardiac Repair. Nat. Commun. 12 (1), 1412. 10.1038/s41467-021-21682-7 33658506 PMC7930285

