# Supplementary material for: Low-Stiffness Hydrogels Promote Peripheral Nerve Regeneration Through the Rapid Release of Exosomes
Source: Front Bioeng Biotechnol. 2022 Jun 23;10:922570. doi: 10.3389/fbioe.2022.922570 (PMC9260118; doi:10.3389/fbioe.2022.922570)
Supplement: Supplementary file 1 [file DataSheet1.docx]

## Supplementary Figures


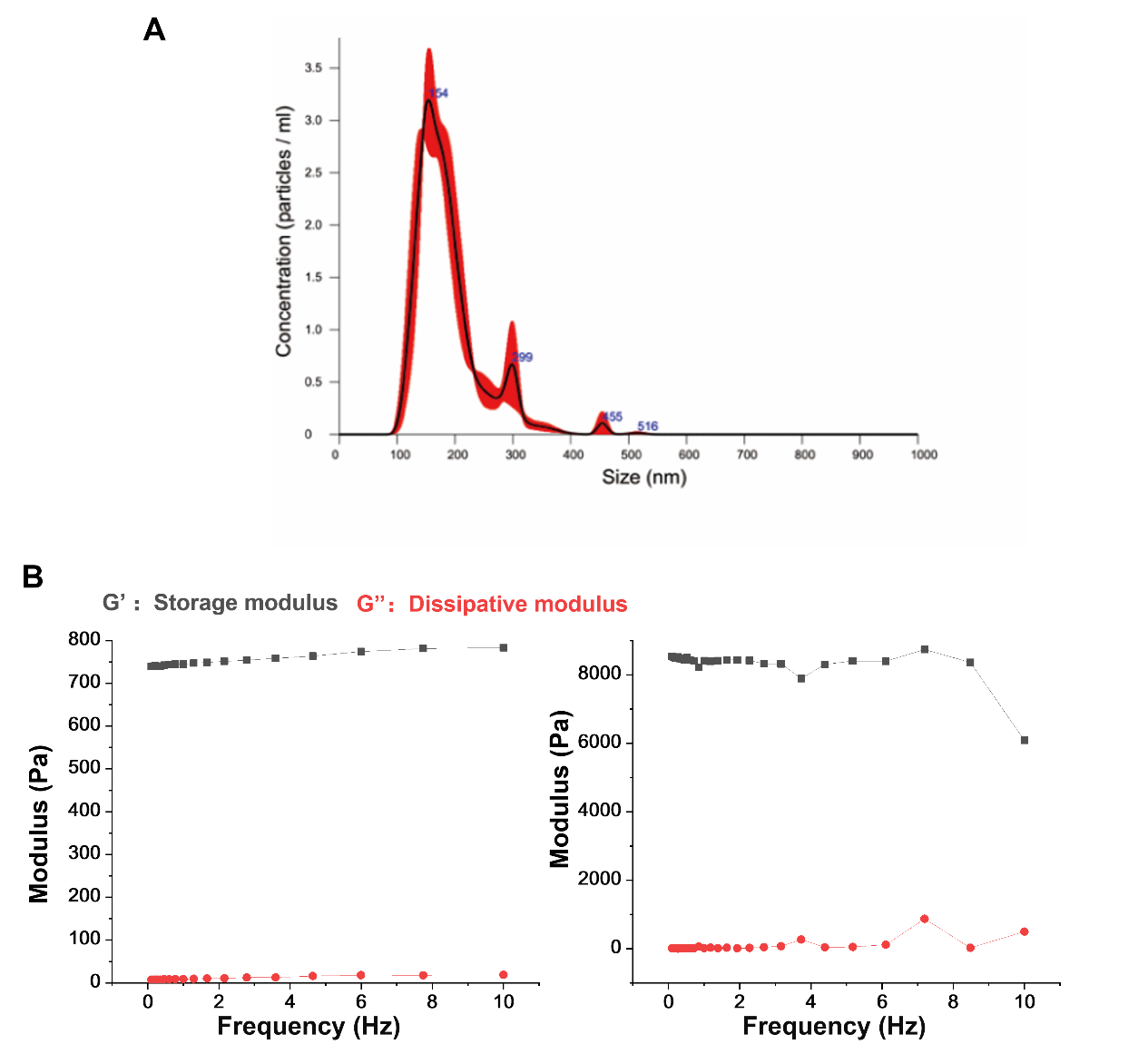


**Figure S1. Exosomes and hydrogel characterization. A**, The size of exosomes derived from mesenchymal stem cells was characterized by Nanoflow. **B**, Fluorescence 3D imaging of hydrogels loaded with PKH26 labeled exosomes (red: PKH26, bar=50μm). **C**, Characterization of the mechanical properties of hydrogels with different concentrations using a rotational rheometer (left: 40 μg/μl; right: 100 μg/μl).


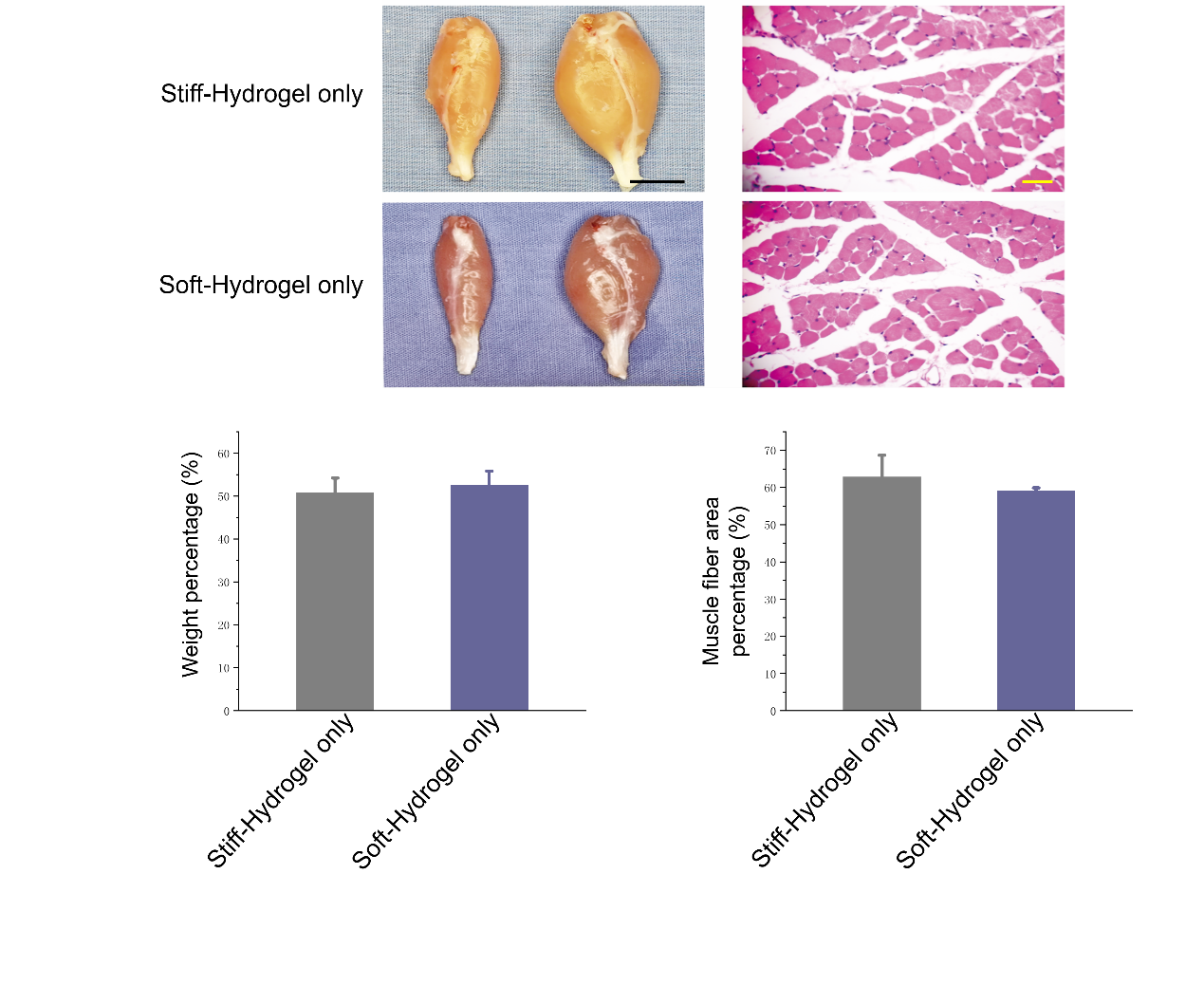


**Figure S2.** **There is no significant difference in the repair effects of single hydrogel components with different stiffness on sciatic nerve injury.** Photographs of rat gastrocnemius muscles on the operative and non-operative sides and HE stained images of the transverse section of the gastrocnemius muscle on the operating side (above, bar=1cm black, bar=100μm yellow). Statistics show that different stiffness hydrogel components have no significant difference in repairing effects on muscle atrophy after sciatic nerve injury ( Weight *p*=0.53205; area *p*=0.33823; n=3).


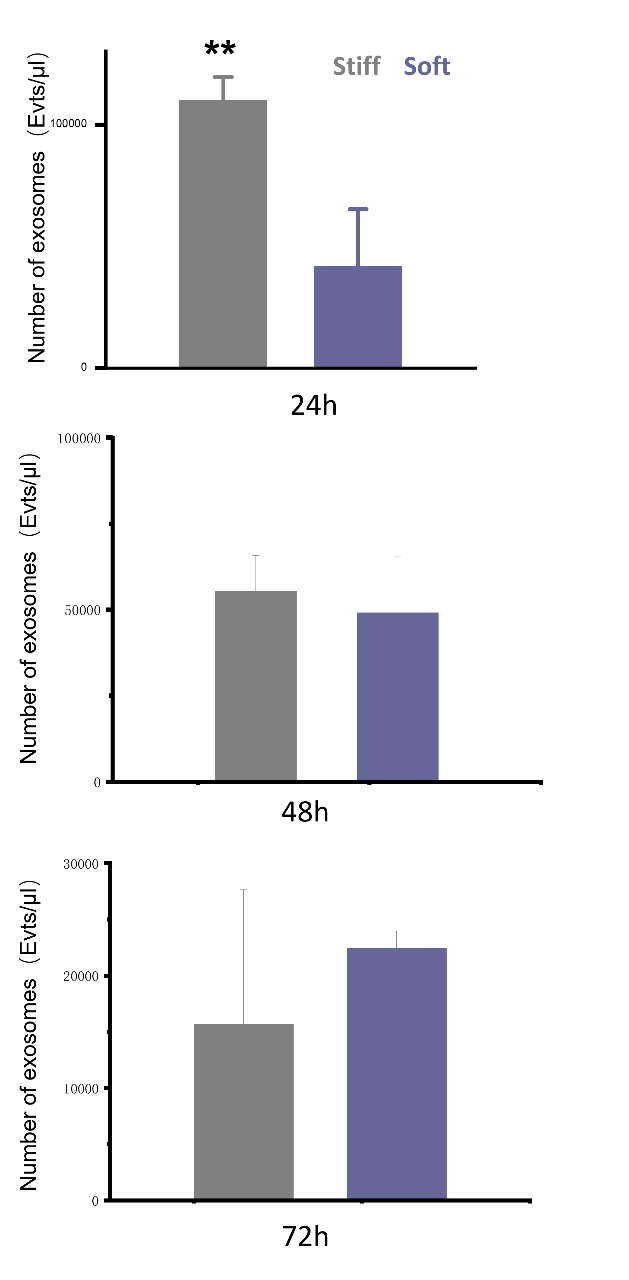


**Figure S3.** The hydrogels of different hardness were loaded with exosomes and incubated with PBS. After 24 hours, 48 hours and 72 hours, the PBS was collected, and the number of exosomes released into the PBS from the gel was characterized by Nanoflow. Stiff hydrogel release exosomes significantly faster than soft hydrogel at 24 hours (24 hours *p* =0.00948, 48 hours p=0.6927, 72 hours p=0.38797; n=3).


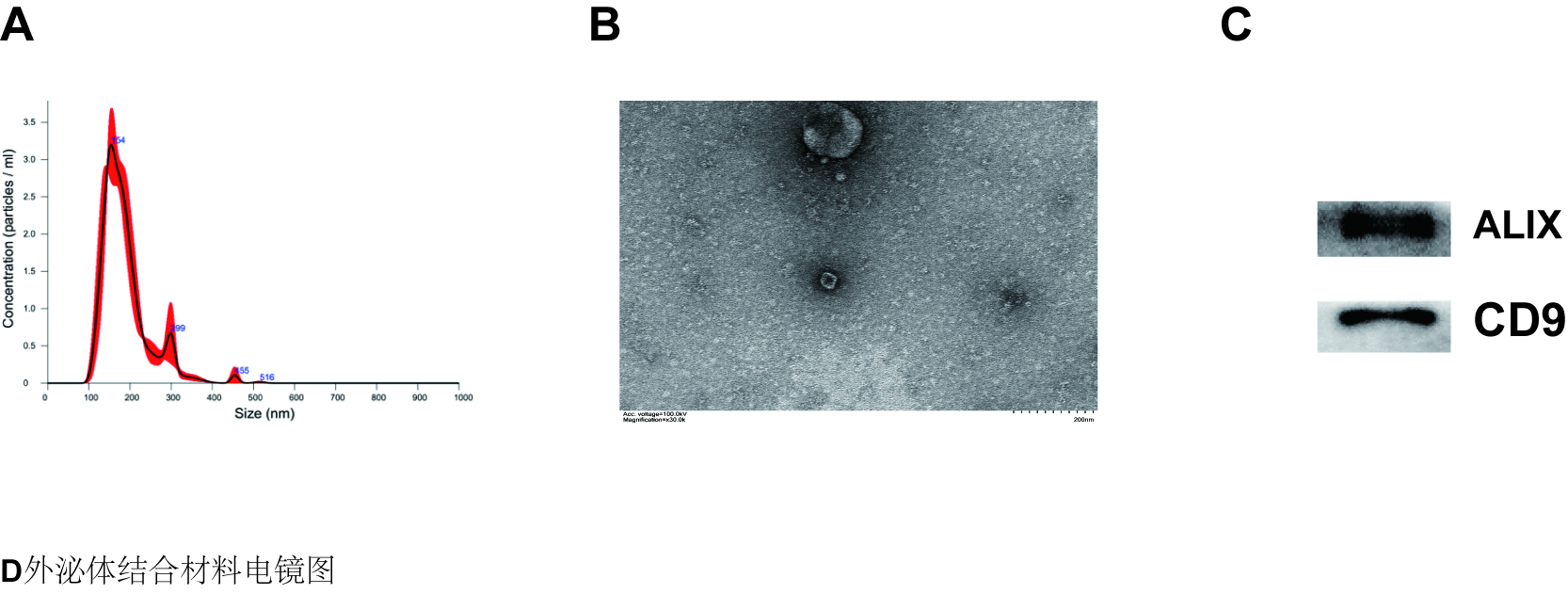


**Figure S4.** TEM of exosomes.


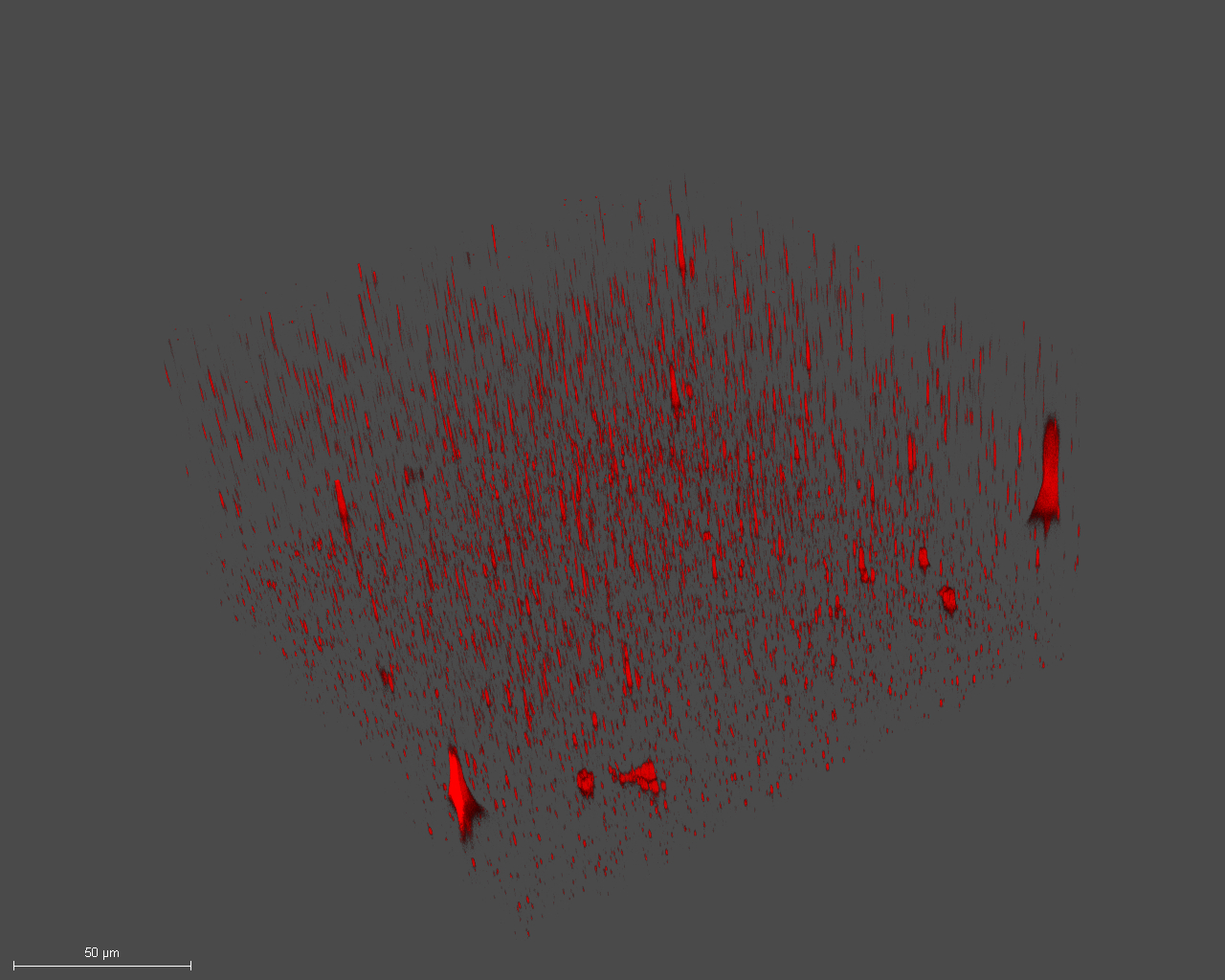


**Figure S5.** Fluorescence 3D imaging of hydrogels loaded with PKH26-labeled exosomes.
